# Biofilm Biology and Engineering of *Geobacter* and *Shewanella* spp. for Energy Applications

**DOI:** 10.3389/fbioe.2021.786416

**Published:** 2021-12-03

**Authors:** Yidan Hu, Yinghui Wang, Xi Han, Yawei Shan, Feng Li, Liang Shi

**Affiliations:** ^1^ Department of Biological Sciences and Technology, School of Environmental Studies, China University of Geosciences, Wuhan, China; ^2^ Key Laboratory of Systems Bioengineering (Ministry of Education), SynBio Research Platform, Collaborative Innovation Center of Chemical Science and Engineering, School of Chemical Engineering and Technology, Tianjin University, Tianjin, China; ^3^ State Key Laboratory of Biogeology and Environmental Geology, China University of Geosciences, Wuhan, China; ^4^ Hubei Key Laboratory of Yangtze Catchment Environmental Aquatic Science, China University of Geosciences, Wuhan, China; ^5^ State Environmental Protection Key Laboratory of Source Apportionment and Control of Aquatic Pollution, Ministry of Ecology and Environment, Wuhan, China

**Keywords:** *Geobacter*, *Shewanella*, exoelectrogen, biofilm, biofilm engineering, microbial fuel cells

## Abstract

*Geobacter* and *Shewanella* spp. were discovered in late 1980s as dissimilatory metal-reducing microorganisms that can transfer electrons from cytoplasmic respiratory oxidation reactions to external metal-containing minerals. In addition to mineral-based electron acceptors, *Geobacter* and *Shewanella* spp. also can transfer electrons to electrodes. The microorganisms that have abilities to transfer electrons to electrodes are known as exoelectrogens. Because of their remarkable abilities of electron transfer, *Geobacter* and *Shewanella* spp. have been the two most well studied groups of exoelectrogens. They are widely used in bioelectrochemical systems (BESs) for various biotechnological applications, such as bioelectricity generation *via* microbial fuel cells. These applications mostly associate with *Geobacter* and *Shewanella* biofilms grown on the surfaces of electrodes. *Geobacter* and *Shewanella* biofilms are electrically conductive, which is conferred by matrix-associated electroactive components such as *c*-type cytochromes and electrically conductive nanowires. The thickness and electroactivity of *Geobacter* and *Shewanella* biofilms have a significant impact on electron transfer efficiency in BESs. In this review, we first briefly discuss the roles of planktonic and biofilm-forming *Geobacter* and *Shewanella* cells in BESs, and then review biofilm biology with the focus on biofilm development, biofilm matrix, heterogeneity in biofilm and signaling regulatory systems mediating formation of *Geobacter* and *Shewanella* biofilms. Finally, we discuss strategies of *Geobacter* and *Shewanella* biofilm engineering for improving electron transfer efficiency to obtain enhanced BES performance.

## Introduction

Dissimilatory metal-reducing microorganisms (DMRM) can oxidize organic matters or hydrogen and then transfer the derived electrons to extracellular metal-containing minerals for respiration under anoxic conditions (Lovley et al., 1987; Lovley and Phillips, 1988). DMRM, ranging from Gram-negative and positive bacteria to archaea, inhabit various environments where they directly contribute to biogeochemical cycling of carbon and metals, such as iron (Fe) and manganese (Mn) (Lovley and Phillips, 1988; Myers and Nealson, 1988; Lovley et al., 1989). *Geobacter metallireducens* and *Shewanella oneidensis* were the first isolated organisms that were unequivocally shown the ability to gain energy for their growth through Fe(III) or Mn(IV) reduction (Lovley et al., 1987; Myers and Nealson, 1988). Since these pioneering studies in late 1980s, it has been found that most organisms conserving energy to support their growth by the reduction of Fe(III) or Mn(IV) fall within *Geobacter* spp. that predominate over other DMRM and play important roles in the oxidation of organic compounds in subsurface environments. On the other hand, the Gram-negative bacteria *Shewanella* spp. are facultative anaerobic microbes capable of using a broad range of electron donors and acceptors. They are found in different ecological niches, such as marine and freshwater ecosystems (Heidelberg et al., 2002). Over the past 40 years, thorough and rigorous studies have made *Geobacter* and *Shewanella* spp. the two widely investigated groups of DMRM.

*Geobacter* and *Shewanella* spp. have been of scientific interest not only due to their involvement in a range of biogeochemical cycles, but also the potential of harnessing their electron transfer activities for a range of biotechnological applications (Lloyd et al., 2003). For example, in addition to mineral-based acceptors, *G. metallireducens* can transfer electrons directly to methanogens, such as *Methanotrix harundinacea* and *Methanosarcina barkeri*, for methane production in wetlands and anaerobic digesters (Summers et al., 2010; Rotaru et al., 2014). This process is referred to as direct interspecies electron transfer that permits the conversion of wastes to methane. Another broadly studied attributes of *Geobacter* and *Shewanella* spp. are their capability to transfer electrons to or accept electrons from electrodes (Bond and Lovley, 2003; Gregory et al., 2004; Ueki et al., 2018). Microorganisms with the ability of exocellular electron transfer to electrodes are known as exoelectrogens (Logan, 2009; Logan et al., 2019). Meanwhile, they also have been defined as electrochemically active bacteria (Chang et al., 2006), anode respiring bacteria (Rittmann et al., 2008) and electricigens (Lovley, 2006a). This group of microorganisms are widely used in bioelectrochemical systems (BESs) for various biotechnological applications (Nealson and Rowe, 2016; Kumar et al., 2017). For example, exoelectrogens show promise as biocatalysts for efficient conversion of a wide range of organic wastes and renewable biomass to electricity (i.e., microbial fuel cells, MFCs) (Rabaey and Verstraete, 2005; Lovley, 2006b; Logan, 2009). It should be pointed out that the highest current densities to date are produced by mixed cultures that are often dominated by *Geobacter* spp. (Koch and Harnisch, 2016; Logan et al., 2019). Moreover, *Geobacter sulfurreducens* KN400 generated one of the highest known power densities in pure cultures (Yi et al., 2009), highlighting the promise of *Geobacter* spp. in energy applications. Thus, *Geobacter* and *Shewanella* spp. have been used in BESs for various environmental and energy applications (Koch and Harnisch, 2016; TerAvest and Ajo-Franklin, 2016; Li et al., 2018; Logan et al., 2019).

Most biotechnological applications of *Geobacter* and *Shewanella* spp. associate with the formation and conductivity of *Geobacter* and *Shewanella* biofilms (Hu et al., 2019; Mukherjee et al., 2020). A biofilm is a well-organized 3-dimensional (3D) architecture in which cells are enclosed by a matrix of self-produced extracellular polymeric substances (EPS) (Wingender et al., 1999). *Geobacter* and *Shewanella* biofilms are electrically conductive, which is conferred by matrix-associated electroactive components such as *c*-type cytochromes (*c*-Cyts) and nanowires (Borole et al., 2011; Bond et al., 2012; Snider et al., 2012; Xu et al., 2018). The electroactivity of these redox molecules permits the cells to grow at a distance from electrodes. To improve *Geobacter* and *Shewanella* biofilm-mediated bioprocesses for various biotechnological applications, engineering their biofilms is a promising strategy.

The state-of-the-art understanding of biofilm biology has enabled biofilm engineering for desirable performances in practical applications. This review first briefly discusses extracellular electron transfer of *Geobacter* and *Shewanella* and compares the roles of planktonic and biofilm-forming cells in BESs. We then review biofilm biology with the focus on biofilm development, biofilm matrix, heterogeneity in biofilm and signaling regulatory systems that mediate formation of *Geobacter* and *Shewanella* biofilms. Finally, we discuss strategies of biofilm engineering for improving electron transfer performances of *Geobacter* and *Shewanella* biofilms in BESs.

## Extracellular Electron Transfer of *Geobacter* and *Shewanella*: Biofilm vs. Planktonic Exoelectrogens

**FIGURE 1:**
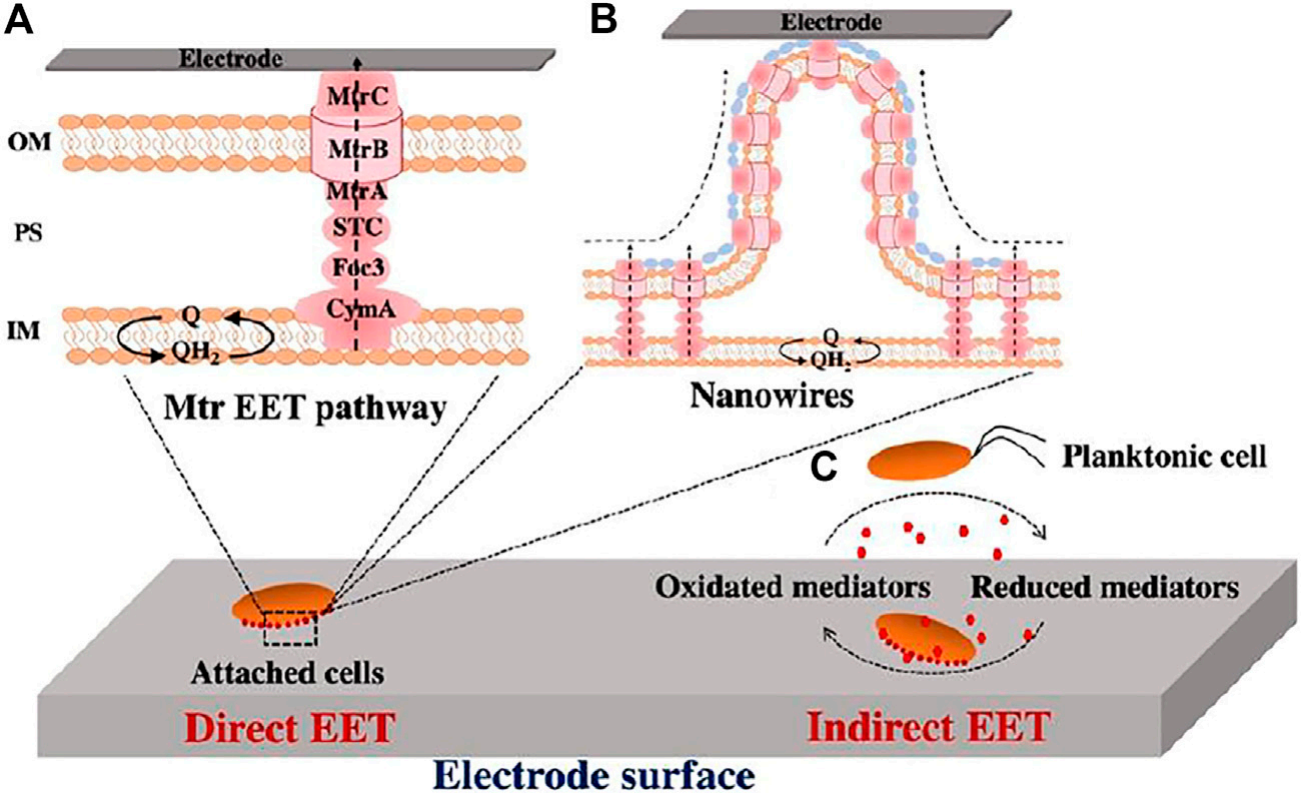
Extracellular electron transfer (EET) pathways of *Shewanella oneidensis* MR-1 to electrodes. **(A)** Direct electron transfer via Mtr pathway. **(B)** Direct electron transfer via the outer membrane and periplasm extensions (previously described as nanowires). **(C)** Indirect electron transfer via electron shuttle flavins. Q, quinone, QH_2_, quinol, IM, inner membrane, PS, periplasmic space, OM, outer membrane.

Microbial electron exchange with extracellular substrates, such as electrodes in BESs, is often called microbial extracellular electron transfer (EET). The most well characterized microbial EET pathway is the Mtr pathway of *S. oneidensis* MR-1. *S. oneidensis* MR-1 transfers electrons from the quinone/quinol pool in the cytoplasmic membrane to the bacterial surface through redox and structural proteins, including the inner membrane *c*-Cyt CymA, the periplasmic *c*-Cyts Fcc3 and STC, and the outer membrane porin-*c*-Cyt complex MtrCAB (Figure 1A) (Nealson et al., 2002; Shi et al., 2016; Jiang et al., 2019). Eventually, electrons are transferred from the bacterial surface to extracellular acceptors directly *via* the outer membrane *c*-Cyts or *via* nanowires (Figures 1A,B), or indirectly *via* self-secreted electron mediator flavins (Figure 1C) (Bretschger et al., 2007; Marsili et al., 2008; Meitl et al., 2009; Coursolle et al., 2010; Kotloski et al., 2013; Okamoto et al., 2013; Xu et al., 2018). *Shewanella* nanowires are the extensions of the outer membrane and periplasm in which the cell surface *c*-Cyts MtrC and OmcA are the key players for long-distance electron transfer (Gorby et al., 2006; Pirbadian et al., 2014). Another widely studied EET pathway is Pcc EET pathways of *G. sulfurreducens*. Like Mtr pathway of *S. oneidensis* MR-1, Pcc EET pathways transfer electrons from quinone/quinol pool in the cytoplasmic membrane to the bacterial surface using a series of multiheme *c*-Cyts (Liu Y. et al., 2014; Shi et al., 2014; Liu Y. et al., 2015; Shi et al., 2016). It should be noted that nanowires produced by *G. sulfurreducens* are important for long-range EET (Reguera et al., 2006; Reguera, 2018; Lovley and Walker, 2019). *G. sulfurreducens* nanowires were previously thought to be electrically conductive type IV Pili assembled by PilA protein. (Reguera et al., 2005; Malvankar and Lovley, 2014; Lovley, 2017; Tan et al., 2017; Lovley and Walker, 2019; Malvankar et al., 2012b). However, it is based on genetic studies that *pilA* mutant did not produce filaments (Reguera et al., 2005), which is lack of a direct evidence. By using cryo-electron microscopy with atomic force microscopy, Wang et al. (2019), Yalcin et al. (2020) determined composition and structure of nanowires and found that nanowires are polymerized by *c*-Cyts OmcS and OmcZ. They further correlated their protein structures with functions including their conductivity and stiffness. The results revealed that OmcS nanowires show 100-fold higher conductivity than noncytochrome filaments, whereas OmcZ nanowires exhibit 1000-fold higher conductivity and 3-fold higher stiffness than OmcS nanowires. Moreover, they recently demonstrated the conductivity of pili is very low based on the predicted and observed results, as well as found that OmcS and OmcZ for Δ*pilA*-*N* cells remain in the periplasm rather than extracellular fraction. Thus, they proposed that *Geobacter* pili exhibit secretory behaviour (Gu et al., 2021). On the other hand, a recent study by Liu et al. (2021) demonstrated that *G. sulfurreducens* produces abundant electrically conductive pili but expresses few OmcS-based filaments by directly examining filaments emanating from cells. The debate on the composition of nanowires and their role in long-range electron transfer continues. Moreover, self-secreted flavins contribute to *Geobacter* EET as cofactors by binding to outer membrane *c*-Cyts when electrodes are used as electron acceptors (Okamoto et al., 2014a; Okamoto et al., 2014b; Huang et al., 2018; Thirumurthy and Jones, 2020). Based on these studies, two microbial EET pathways have been proposed: direct EET *via c*-Cyts or electrically conductive nanowires and indirect EET *via* exogenous or endogenous electron shuttles.

The exoelectrogens grown in BESs display two microbial lifestyles, i.e., biofilms on anodes and planktonic cells in anode chambers. Both biofilm cells and planktonic cells can transfer electrons to electrodes through direct EET or indirect EET (Marsili et al., 2008; Harris et al., 2010; Borole et al., 2011). Previous studies showed that *S. oneidensis* MR-1 can form a biofilm on the anode in lactate-fed MFCs (McLean et al., 2010; Watson and Logan, 2010; Roy et al., 2014), and both planktonic cells and biofilms synergistically contribute to current generation (Biffinger et al., 2007). In contrast, the replacement of the medium surrounding biofilm-coated electrodes rarely affects the electron transfer of *Geobacter* spp., indicating that indirect EET mediated by electron shuttles is not involved in the current generation of *Geobacter* MFCs (Bond et al., 2002; Bond and Lovley, 2003). The level of flavins in planktonic cultures of *G. sulfurreducens* is too low to function as electron shuttles for respiration (Okamoto et al., 2014a; Okamoto et al., 2014b). Moreover, characterization of metabolism in *G. sulfurreducens* by constraint-based modeling revealed that EET by direct contact for *G. sulfurreducens* has an energetic advantage over electron shuttling by flavins (Mahadevan et al., 2006). Thus, *G. sulfurreducens* requires direct electrical contact with the surface of the anode electrode for electron transfer (Bond and Lovley, 2003; Reguera et al., 2006). Additionally, previous studies showed that direct EET likely enables higher kinetic rates of electron transfer than the process solely depending on diffusion of electron mediators (Marsili et al., 2008; Srikanth et al., 2008; Marsili et al., 2010). Thus, the ability of an exoelectrogen to form a biofilm is one of key factors for increasing current generation in MFCs.

## Biofilm Biology of *Geobacter* and *Shewanella*

### Biofilm Development

The life cycle of a biofilm is a continuous, highly dynamic process. Biofilms develop through several stages including microbial attachment on surfaces or interfaces, biofilm maturation and biofilm dispersal (Costerton, 1999; O'Toole et al., 2000; Watnick and Kolter, 2000; Flemming et al., 2016). Similar to other biofilm-forming bacteria, exoelectrogens first physically contact and attach to the surfaces of external electron acceptors, such as metal particles, electrode surfaces, or partner bacteria. Attachment stages of *Geobacter* and *Shewanella* biofilms are mediated by functional flagellar system, type IV pilus system and extracellular DNA (eDNA) (Thormann et al., 2004; Gödeke et al., 2011a; Gödeke et al., 2011b; Cologgi et al., 2014; Bennett et al., 2016). Then, biofilms become mature, during which cells form a fixed EPS matrix (Allison, 2003; Flemming and Wingender, 2010). Different from that of nonelectrogenic bacteria, the biofilm matrix of *Geobacter* and *Shewanella* spp. is electrically conductive and contains extracellular electron transfer components, such as cytochromes and other electron mediators (Borole et al., 2011; Reguera, 2018; Xu et al., 2018). This is required for the growth of daughter cells or new cell layers at a distance from external electron accepters. The final stage of a biofilm life cycle is biofilm dispersal in which the planktonic cells are released from a mature biofilm, migrate and attach to new surfaces, and subsequently start a new life cycle (McDougald et al., 2012; Rumbaugh and Sauer, 2020).

### Biofilm Matrix

Cells in a mature biofilm are enclosed in a self-produced EPS matrix that occupies most of biofilm biomass (Allison, 2003). EPS matrix is composed of soluble, gel-forming components such as polysaccharides, proteins and extracellular DNA (eDNA) (Flemming and Wingender, 2010), as well as insoluble components including cellulose, amyloid, flagella and pili (Hobley et al., 2015). By filling and shaping the space between the cells of biofilms, EPS molecules offer a microenvironment for biofilm cells and provide a structural scaffold for biofilm stability (Persat et al., 2015). EPS matrix may also form an external digestion system due to its ability to accumulate enzymes (Tielen et al., 2010). These enzymes, such as protein-, polysaccharide-, oligosaccharide-, or lipid-degrading enzymes and phosphomonoesterases, can degrade diverse matrix components or other nutrients (Allison, 2003; Flemming and Wingender, 2010). After degradation, the products in close proximity to cells are efficiently captured and taken up by cells. Another important role of EPS matrix is to serve as a protective shield against toxins, antimicrobials and desiccation (Flemming and Wingender, 2010). Thus, the matrix is a functionally crucial component of microbial biofilms.

**FIGURE 2:**
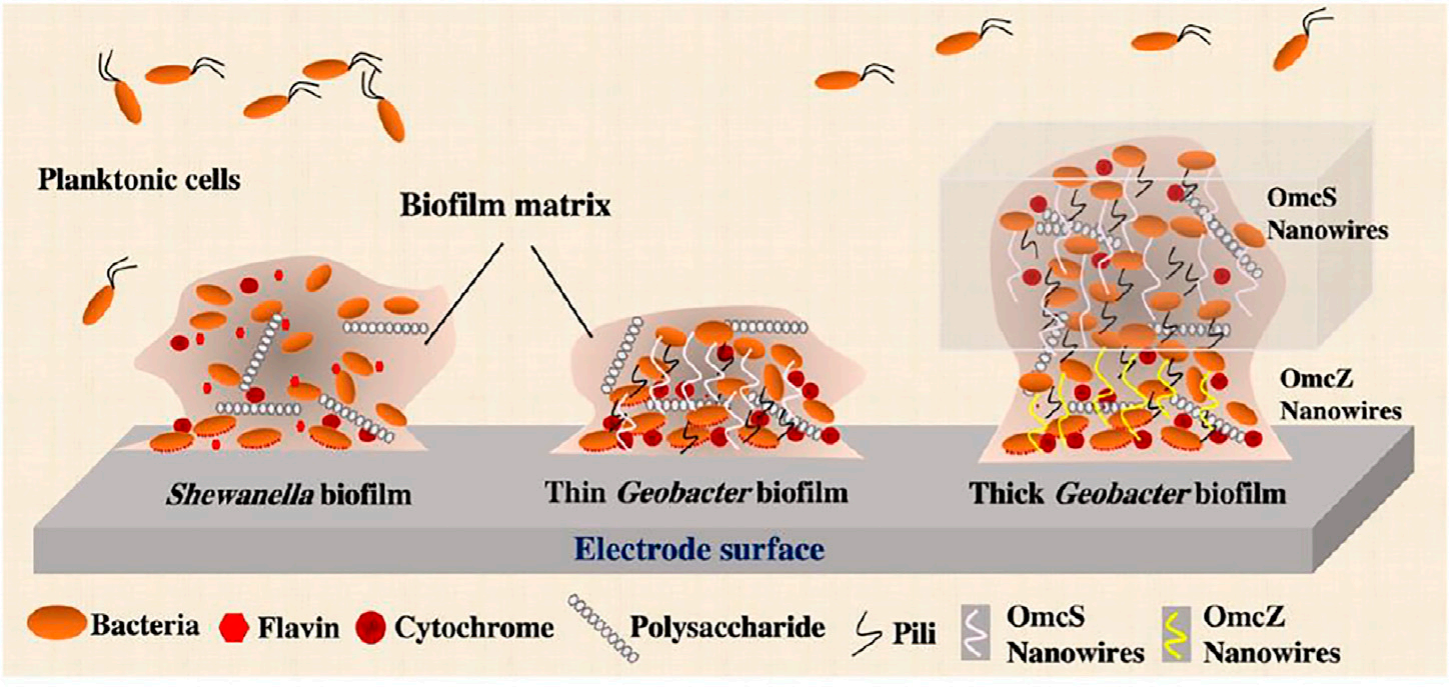
The complex structure of *Shewanella* and *Geobacter* biofilms in a bioelectrochemical system. Microcolonies in the mature biofilm are characterized by an extracellular polymeric substances (EPS) matrix consisting of polysaccharides, proteins and extracellular DNA (eDNA). Different from that of nonelectrogenic bacteria, the biofilm matrix of exoelectrogens is electrically conductive and contains extracellular electron transfer components, such as cytochromes and other electron mediators in *Shewanella* EPS matrix, as well as cytochromes, pili and OmcS/OmcZ-based nanowires in *Geobacter* EPS matrix. Because of higher abundance of cytochromes that form conductive nanowires, *Geobacter* biofilm is much more conductive and thicker than *Shewanella* biofilm.

*Shewanella* matrix contains extracellular polysaccharides, proteins and eDNA (Figure 2) (Mukherjee et al., 2020). Proteomics analysis of EPS extracted from *Shewanella* sp. HRCR-1 biofilm found the presence of 58 extracellular and outer membrane proteins, such as redox active proteins MtrC and OmcA (Cao et al., 2011). The presence of *c*-Cyts in the biofilm matrix of *S. oneidensis* MR-1 is further confirmed by a recent study that detected the electrochemical activities and redox peaks of *c*-Cyts using voltammetry measurement (Xiao et al., 2017). This study also clearly showed the redox peaks of flavins in EPS of *S. oneidensis* MR-1. The EPS matrix of *Shewanella* biofilm likely affects extracellular electron transfer through biofilm *via* redox proteins and electron shuttles.

It has been reported that *Geobacter* spp. are among the major phylotypes in the attached fraction of environment samples (Dong et al., 2021). *Geobacter* biofilms have an electroactive EPS matrix with matrix-associated *c*-Cyts and pili that spatially organize cells well for growth and electron transfer to mineral oxides and electrodes (Figure 2). The studies that showed the reversible reduction and oxidation of the cytochromes demonstrated their functionality as electron carriers for electron transfer in electricity-producing *G. sulfurreducens* biofilms (Liu et al., 2011; Liu and Bond, 2012). The roles of outer membrane cytochromes in biofilm electroactivity were further investigated by the studies that constructed diverse cytochrome-defective mutants and tested their current outputs. Deleting outer membrane *c*-Cyt gene *omcE* or *omcB* from the *G. sulfurreducens* genome has no effect on electricity outputs of MFCs (Holmes et al., 2006; Nevin et al., 2009; Richter et al., 2009). Outer membrane *c*-Cyt OmcS is required for optimum current production in fuel cells in which electrochemical restrictions limit electricity outputs (Holmes et al., 2006), whereas deleting *omcS* has no impact on current production when there is no electrochemical limitation (Nevin et al., 2009). However, the genetic deletion of OmcZ leads to a significant reduction in biofilm electroactivity and electricity output (Nevin et al., 2009; Richter et al., 2009). OmcZ preferentially locates at or closer (<10 µm) to the electrode surface in the anodic *G. sulfurreducens* biofilm (Inoue et al., 2011), suggesting a critical role of OmcZ in the last step of EET, i.e., electron transfer from biofilms to electrodes. Moreover, a recent study discovered that the production of OmcZ can be simulated by electric field (Yalcin et al., 2020). OmcZ has been identified to be an essential matrix-associated *c*-Cyt for electron transfer in thick biofilms. Other matrix-associated *c*-Cyts may be required, but compensatory effects observed in encoding gene-defective strains make their genetic identification challenging (Kim et al., 2005; Mehta et al., 2005). For example, the compensatory effect of OmcZ nanowires probably makes Δ*omcS G. sulfurreducens* biofilms show high current production when the biofilms mature. Thus, OmcZ and OmcS nanowires may have overlapping roles in *G. sulfurreducens* MFC performance (Nevin et al., 2009). Based on these previous studies, Yelcin et al. proposed a model that OmcS nanowires play an important role in EET during the early stage of biofilm growth, while the high conductivity of OmcZ nanowires enables *G. sulfurreducens* to form thick biofilms on the electrode surface of MFC (Figure 2) (Yalcin and Malvankar, 2020). On the other hand, Steidl et al. (2016) found that *G. sulfurreducens* pili have critical impacts on the formation of relatively thick (>10 µm) biofilms on the electrodes.

### Heterogeneity in *Geobacter* and *Shewanella* Biofilms

Steep chemical gradients exist in the EPS matrix of microbial biofilms (Chang et al., 2015). The microbial cells living in these heterogenous environments display different metabolic activities for their adaption to different local environmental conditions by expressing distinct set of genes (Stewart and Franklin, 2008). Cao et al. monitored the spatiotemporal metabolic responses of metabolically active *S. oneidensis* MR-1 biofilms to contaminant exposure, which demonstrated that metabolic responses were spatially stratified based on local microenvironments (Cao et al., 2012). In an aerobic heterotrophic biofilm, organisms are stratified based on oxygen and nutrient availabilities, which become depleted in the lower layers of the biofilm when the consumption rates of oxygen and nutrients in the upper layers of the biofilm are faster than their diffusion rates (Stewart, 2003). The cells in the upper layers of the biofilm often have higher metabolic activities because they have optimal access to nutrients and electron acceptors.

Unlike a biofilm on a surface, the cells in the upper layers of the anode-respiring biofilm have optimal access to soluble electron donors, nutrients and buffering agents, whereas cell layers close to electrodes have optimal access to electron acceptors (Bond et al., 2002). Optimizing electrode-respiring biofilm systems for practical applications greatly requires the understanding of their internal chemical and electrical fluxes including nutrient diffusion, electron transfer and the movement of protons and pH-buffering compounds (Renslow R. S. et al., 2013). Babauta et al. used microelectrodes to quantify pH and redox potential variations in electrode-respiring *S. oneidensis* MR-1 and *G. sulfurreducens* biofilms (Babauta et al., 2011; Babauta et al., 2012). Their results demonstrated that observable pH variation was not detected, and that the redox potential decreased toward the bottom of the biofilm in the *S. oneidensis* MR-1 biofilm. It implies that there is no proton transfer limitation in *S. oneidensis* MR-1 biofilms. On the other hand, they found that pH continued to decrease in the *G. sulfurreducens* biofilm through different growth phase, suggesting that pH was not always a limiting factor for biofilm growth. Physiological stratification in electricity-producing *G. sulfurreducens* biofilms was further investigated by the study showing that the progressive accumulation of cells did not contribute to current production after a maximal current was achieved (Schrott et al., 2014). Furthermore, Chadwick et al. revealed metabolic stratification within current-producing *G. sulfurreducens* biofilms using stable isotope probing coupled to nanoscale secondary ion mass spectrometry (nanoSIMS) (Chadwick et al., 2019). The results demonstrated that cells at the anode surface were most active, and that cell activity decreased with an increase in distance from electrodes. This work also found that the cells nearest the electrode could keep their maximum growth rate in 80-μm-thick biofilms, suggesting that nutrient or buffer diffusion into the biofilm was not rate-limiting. In another interesting study, azo dye was used as an alternative electron acceptor in MFCs with *Shewanella decolorationis* (Yang et al., 2015). They observed electron acceptor-dependent physiological stratification within anode biofilm in the presence of multiple electron acceptors. Collectively, these findings demonstrate the physicochemical and physiological stratifications within electrode-respiring biofilms of *Geobacter* and *Shewanella* spp. It helps improve understanding of the electron transfer mechanism within anode-respiring biofilm.

### Cyclic Dinucleotide Signaling System in the Development of *Geobacter* and *Shewanella* Biofilms

**FIGURE 3:**
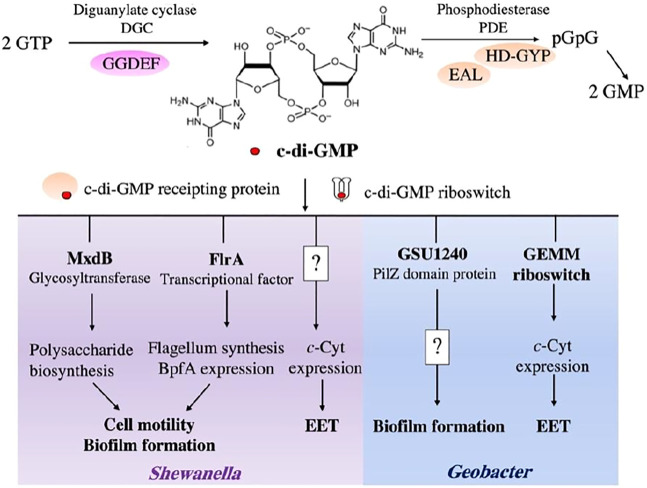
c-di-GMP signaling in *Shewanella* and *Geobacter* cells. The intracellular level of c-di-GMP is mediated by DGCs with GGDEF domains and PDEs with EAL/HD-GYP domains. In *Shewanella* spp., the c-di-GMP signaling network coordinates the biosynthesis of extracellular polysaccharides through interaction with MxdB glycosyltransferase, a polysaccharide biosynthetic enzyme. Flagellum synthesis and BpfA expression are controlled by c-di-GMP through its responsive transcriptional factor, FlrA. c-di-GMP also positively regulates *c*-cytochrome (*c*-Cyt) expression in *S. oneidensis* MR-1, but molecular mechanism is unclear. In *Geobacter* spp., c-di-GMP regulates biofilm formation and *c*-Cyt expression using PilZ domain-containing protein and GEMM riboswitches, respectively. How c-di-GMP regulates formation of *Geobacter* biofilm is currently unknown.

Bis-(3′-5′)-cyclic dimeric GMP (c-di-GMP) is a key regulator in biofilm formation (Hengge, 2009). It is respectively synthesized and degraded by diguanylate cyclases (DGCs) containing GGDEF domain and phosphodiesterases (PDEs) harboring EAL/HD-GYP domain (Figure 3) (Jenal et al., 2017). The dynamic change of c-di-GMP level is converted ultimately into specific cellular responses by mediating the responsive effectors, such as riboswitches and c-di-GMP receipting proteins (Römling et al., 2005; Hengge, 2009).

Environmental cues, such as nitric oxide, affect biofilm formation through the turnover of c-di-GMP signaling network, which has been discovered in both *Shewanella woodyi* and *S. oneidensis* (Liu et al., 2012; Plate and Marletta, 2012). It indicates that c-di-GMP is a crucial intracellular regulator for controlling formation of *Shewanella* biofilm. Moreover, a high abundance of genes encoding DGCs and PDEs in *Shewanella* genomes further emphasize the importance of c-di-GMP signaling network in the regulation of physiological processes of *Shewanella* spp. (Mukherjee et al., 2020). For example, *S. oneidensis* are predicted to carry 51 GGDEF-containing proteins, 27 EAL-containing proteins, and 20 proteins that contain both GGDEF and EAL/HD-GYP. In *Shewanella* spp., c-di-GMP mediates biofilm formation by interacting with polysaccharide biosynthetic enzymes (e.g., MxdB glycosyltransferase) to activate the biosynthesis of extracellular polysaccharides (Gambari et al., 2019). Previous study showed that c-di-GMP-responsive transcriptional factor FlrA controls the expression of genes for the synthesis of flagella and an adhesin protein BpfA (Figure 3) (Cheng et al., 2017; Gao et al., 2018). In addition to mediating the biofilm development, a recent study showed that c-di-GMP signaling network regulates the expression of *c*-Cyts, such as CymA, OmcA and MtrCAB, in *S. oneidensis* MR-1, which provides new insights into the physiological functions of c-di-GMP in *Shewanella* spp. (Ng et al., 2020) (Figure 3). Notably, a cyclic adenosine 3′,5′-monophosphate (cAMP) also commonly exists in *Shewanella* spp. Previous study demonstrated that cAMP-CRP (cyclic adenosine 3′,5′-monophosphate receptor protein) regulatory system regulates multiple bidirectional EET-related pathways (Saffarini et al., 2003; Charania et al., 2009). Collectively, cyclic dinucleotides (CDNs) regulate both *Shewanella* biofilm formation and the expression of EET-related genes or pathways, such as *c*-Cyts and flavin biosynthesis pathway.

Numerous genes that encode GGDEF, EAL, and both GGDEF and EAL/HD-GYP domain-containing proteins are found in *Geobacter* genomes. Deletion of *esnD* gene encoding a DGC reduces c-di-GMP levels in the cells of *G. sulfurreducens*. This disrupts the growth of *G. sulfurreducens* biofilm on −0.1 V vs. SHE electrodes, and decreases the current levels (Hallberg et al., 2019). Leang et al. deleted the gene GSU1240 of *G. sulfurreducens*, which was predicted to encode a protein with the PilZ domain (Figure 3). They found that the GSU1240-deficient mutant formed a highly cohesive and more conductive biofilm and produced 70% higher power densities than the wild-type strain (Leang et al., 2013). Moreover, Tremblay et al. (2011) found that a nucleotide insertion in a GEMM (Genes for the Environment, for Membranes and for Motility) riboswitch of *G. sulfurreducens* increased the expression of its downstream *pgcA* gene. This results in an enhanced Fe(III) reduction rate (Figure 3). GEMM riboswitch is a typical c-di-GMP binding riboswitch. Similar to *G. sulfurreducens*, the *G. metallireducens* genome also contains GEMM riboswitches and a high abundance of genes encoding the proteins with GGDEF, EAL, and both GGDEF and EAL domain (Kellenberger et al., 2015), indicating the important role of c-di-GMP signaling network in *Geobacter* spp. Despite these advances, our understanding of how c-di-GMP impacts the formation of *Geobacter* biofilm is far from complete.

*Geobacter* spp. produce another CDNs, i.e., cyclic AMP-GMP (cGAMP or 3′, 3′-cGAMP). Thirty years ago, the GGDEF domain-containing enzymes were considered as c-di-GMP synthases. However, Hallberg et al. recently found that the hybrid promiscuous GGDEF enzyme GacA specifically produced cGAMP in *G. sulfurreducens* (Hallberg et al., 2019). Moreover, GEMM riboswitches preferentially recognize cGAMP (Kellenberger et al., 2015; Nelson et al., 2015). GEMM riboswitches are conserved upstream of EET-associated genes, such as *omcST*, *pilMNPPQ* and *omcAHG* in *G. sulfurreducens* (Hallberg et al., 2019). These observations suggest a possible role of cGAMP in regulating EET process of *G. sulfurreducens*. Hallberg et al. found that the Δ*gacA* deletion strain of *G. sulfurreducens* was defective in the reduction of insoluble Fe(III) oxide particles, demonstrating the importance of cGAMP in the growth of *G. sulfurreducens* with Fe(III) oxides as electron acceptors (Hallberg et al., 2019).

### Quorum Sensing Systems in the Development of *Geobacter* and *Shewanella* Biofilms

Microbes or microbial communities can synchronize behaviors in response to population density by detecting the accumulation of self-produced extracellular chemical signals. This process is known as quorum sensing (QS). A high cell density allows accumulation of the signals in a local environment to the levels that are required to activate signal-responsive transcriptional factors *via* specific binding. The activated transcriptional factors then regulate the expressions of the genes with QS responsive promoters (Mukherjee and Bassler, 2019; Mukherjee et al., 2020). Quorum sensing occurs in both Gram-positive and negative bacteria *via* a diversity of chemical signals typically classified to acyl homoserine lactones (AHLs), fautoinducer-2 (AI2), diffusible signal factor (DSF) and peptides (Papenfort and Bassler, 2016). Abundant literatures show that QS participates in different stages of biofilm development for different species (O'Toole et al., 2000; Flemming et al., 2016; Rumbaugh and Sauer, 2020).

One of the most well-studied QS system in Gram-negative bacteria are AHL-based QS system that is characterized by two core proteins, the LuxI-type protein (i.e., the AHL synthase), and the LuxR-type protein (i.e., the AHL-responsive transcriptional factor) (Papenfort and Bassler, 2016). LuxI and LuxR homologs were found in *Geobacter uraniireducens* genome (Gelencsér et al., 2012). Chen et al. found that the addition of endogenous AHLs enhanced the electroactivity of a mix-culture biofilm grown on the anodes of MFCs most likely by increasing the relative abundance of electroactive bacteria assigned to *Geobacter* spp. (Chen et al., 2017). The role of AHL signal in a pure culture of *Geobacter* spp. was further investigated. The results showed that the detectable self-secreted AHLs of *Geobacter soli* GSS01 enhanced the formation and electrochemical activity of the biofilm grown on the anode of MFC. Exogenous addition of AHLs further facilitated EPS production (Jing et al., 2019). They also showed that AHL addition increased the biomass, cell viability and EPS abundance of *G. soli* GSS01 biofilm grown on the cathode. Their studies demonstrated, for the first time, the critical role of AHL-type signal in the formation and electroactivity of *Geobacter* biofilm (Jing et al., 2019). Moreover, AHL was detected in cocultured aggregates of *G. metallireducens* and *G. sulfurreducens*, indicating AHL-based QS system may regulate *Geobacter* syntrophic growth and aggregation (Wei et al., 2017).

The QS systems in *Shewanella* spp. are well summarized in a recent review (Mukherjee et al., 2020). Briefly, several LuxR homologous genes are predicted in the genomes of *Shewanella* spp. including *S. woodyi* MS32 and *S. baltica* (Li J. et al., 2020; Hayek et al., 2020). The genome of *S. oneidensis* is predicted to contain seven putative LuxR family proteins. However, they are not LuxR-type QS transcriptional factors (Learman, 2008). Different from *Geobacter* spp*.*, detectable AHL signals are rarely present in *Shewanella* spp. (Zhang et al., 2017; Mukherjee et al., 2020). Moreover, there has been no evidence showing the involvement of QS systems in formation of *Shewanella* biofilm.

## *Geobacter* and *Shewanella* Biofilm Engineering for Energy Applications

### EPS Matrix-Targeted Biofilm Engineering for Electricity Generation in MFCs

MFCs show potential to power electronic devices in remote environments such as sea-floors and to remove organic wastes from contaminated areas in conjunction of electricity generation (Lovley, 2006b; Logan, 2009). But most perceived practical applications are limited by the low current output of MFC. The previous study has shown that the conductivity of the biofilm grown on anodes greatly affected *G. sulfurreducens* MFC performance (Malvankar et al., 2012a). The conductivity through *G. sulfurreducens* biofilm is mainly attributed to cytochromes and nanowires that mediate the short- and long-range electron transfer between cells and electrodes (Reguera et al., 2006; Richter et al., 2009; Malvankar et al., 2011). The expression of *G. sulfurreducens* pili in *Pseudomonas aeruginosa* resulted in a dramatically increased bioelectricity output of *P. aeruginosa* MFCs (Liu et al., 2019a). In contrast, Liu et al. expressed *P. aeruginosa* type IV Pili into *G. sulfurreducens*, resulting in the decreased electricity output (Liu X. et al., 2014). Yi et al. used a selective strategy to obtain a strain of *G. sulfurreducens* KN400 that showed a lower internal resistance and generated a higher electricity output than *G. sulfurreducens* DL-1. The increased electricity output is due to the increased production of pili (Yi et al., 2009). Additionally, the expression of flagella enables *G. sulfurreducens* KN400 to form a thicker biofilm on the anode as compared to *G. sulfurreducens* DL-1 without flagella. Although they are nonconductive, flagella also contribute to the conductivity of *G. sulfurreducens* biofilm. Liu and his collaborators found that flagella can act as *Geobacter* biofilm scaffolds to stabilize the biofilm and anchor extracellular cytochromes with an orderly arrangement, which facilitates extracellular electron transfer (Liu et al., 2019b). In a recent study, overexpression of GSU1501 (a gene for ATP-dependent exporter within an operon of polysaccharide biosynthesis) promoted polysaccharide secretion. The enhanced production of polysaccharides stimulated the formation of the biofilm with higher cell viability. This enhanced the current generation of *G. sulfurreducens* MFCs (Zhuang et al., 2020). Collectively, these studies demonstrate that biofilm matrix is a desirable target to engineer *Geobacter* biofilm for improving MFC performance.

### Chemical Signaling-Targeted Biofilm Engineering for Electricity Generation in MFCs

The switch of bacterial lifestyle between planktonic and sessile modes is often mediated by sophisticated signaling networks that control intracellular levels of small molecules, such as c-di-GMP and quorum sensing signaling (Parsek and Greenberg, 2005; Valentini and Filloux, 2016). Therefore, the manipulations of these signaling networks can efficiently engineer biofilms to improve biofilm-based bioprocesses. Liu et al. and Hu et al. enhanced the formation of *S. oneidensis* biofilm on the anodes by overexpressing a DGC YdeH and a near-infrared-light responsive DGC BphS in *S. oneidensis*, respectively (Liu T. et al., 2015; Hu et al., 2017). The overexpression of these two genes enhanced the biofilm formation and increased the electricity generation of *S. oneidensis* MFCs. Cheng et al. functionally expressed the gene for an adenylate cyclase in *S. oneidensis* and used the engineered strain in microbial electrochemical systems (Cheng et al., 2020). The engineered strain exhibited the enhanced bidirectional EET capacities and the enhanced rate of reducing Cr(VI). The biofilm of *G. sulfurreducens* was engineered by the manipulation of c-di-GMP effectors. The deletion of the gene encoding a c-di-GMP binding effector resulted in the formation of a thicker and much more conductive *G. sulfurreducens* biofilm, which subsequently increased the power density of MFCs (Leang et al., 2013).

QS systems are also targeted to engineer *S. oneidensis* biofilms. Hu et al. used LuxR/AHL QS system originated from *Vibrio fischeri* to construct a gene circuit of AND logic gate. This circuit includes three parts: an isopropyl β-D- thiogalactopyranoside (IPTG)-inducible module, a QS module and an output module (Hu et al., 2015). IPTG inducible module is composed of an IPTG inducible promoter to control the expression of the transcriptional factor gene *luxR*. The expressed LuxR is activated by AHL to drive the expression of the gene, i.e., *mtrA*, in the output module. They introduced this gene circuit into *S. oneidensis mtrA*-deletion mutant and applied the engineered strain in MFCs. The presence of both IPTG and AHL activates MtrA expression, which restores the EET pathway and increases electricity output (Hu et al., 2015). In another study, the *lux* QS system was used to construct a population-state decision system for intelligently reprogramming the EET regulation system (Li F.-H. et al., 2020). The EET-related genes or pathways, such as *cymA*, shuttle exporter genes or flavin biosynthesis pathway, were located at the downstream of QS system and could be expressed at a certain population-state threshold. This system permitted the bacterial growth first and then shifted the metabolic flux form bacterial growth to EET. The engineered strain exhibited a higher EET efficiency and an improved pollutant reduction ability (Li F.-H. et al., 2020).

### Artificial Biofilm

Probably because of nutrient diffusion limitation and insufficient interaction between bacteria and electrodes, the thickness of the biofilms on the anode is often limited to several tens of micron (Renslow R. et al., 2013). To circumvent this limitation, the biofilms are prepared by embedding microbial cells in a matrix made of synthetic polymers. The biofilms prepared in this way is often referred to as “artificial electroactive biofilm” (Adachi et al., 2008). The materials for the building up of an artificial electroactive biofilm are required to provide a high cell viability and ensure an efficient electrical contact between cells and materials. Yu et al. (2011) designed an artificial *S. oneidensis* biofilm with a conductive matrix interlocked with the conductive polymer chains of polypyrrole. Similarly, ready-to-use artificial bioelectrodes were constructed through the immobilization of *G. sulfurreducens* cells in composite materials associating with silica gel and carbon felt fibers (Estevez-Canales et al., 2018). In another study by Yong et al. (2014) a self-assembled 3D reduced graphene oxide (rGO)/*S. oneidensis* hybrid biofilm was formed by a “fishing” process where the GO nanosheets serving as nets caught the bacterial “fish.” Compared to a natural biofilm, this artificial electroactive biofilm exhibits a dramatical increase in power densities due to enhanced electrical conductivity of biofilms. However, the further improvement of BES performance is largely limited by sluggish biotic/abiotic interfacial electron exchange between cells and materials. To improve electron exchange between living cells and abiotic surfaces, a recent study developed a single cell electron collector. In this collector, FeS nanoparticles are confined in the periplasm and on the outer membrane surface to *in-situ* build an interconnected intact conductive layer on and cross the individual cell membrane (Yu et al., 2020). A single cell electron collector permits intimate association of the conductive nanoparticles with the cellular electron transfer machinery and maximizes the interfacial area between microbial cells and electrodes, which substantially enhanced high electron transfer rate and BES performance. Similarly, by introducing *trans*-outer-membrane silver nanoparticles, Cao et al. (2021) obtained the highest power density of *Shewanella* MFCs reported to date. Collectively, all of these results demonstrate a promising and new platform for wiring-up cells with abiotic interface.

### Outlook

Biofilm is the prevalent lifestyle of microbial growth in natural and engineered environments (Flemming et al., 2016). The electroactive *Geobacter* and *Shewanella* biofilms have attracted increasing attention from academic and industrial communities because of their promise in various biotechnological applications, such as bioelectricity generation *via* MFCs. However, the low electricity output largely limits the practical applications of MFCs. The improved understanding of their EET molecular mechanisms and biofilm biology have greatly helped *Geobacter* and *Shewanella* biofilm engineering for improving MFC performance. The rapid advance of synthetic biology has also significantly helped the genetical modification of *Geobacter* and *Shewanella* spp. (Leang et al., 2013; Li et al., 2018; Cao et al., 2019). Furthermore, conductive metal nanoparticles have been used to improve bacteria loading capacity and the conductivities of electrodes and microbial cells. However, few studies couple genetically modified living cell catalysis and material catalysts. Thus, future investigation should focus on the genetically engineered cell-nanoparticle hybrid systems to further push the limits of MFCs.

Most studies of *Geobacter* and *Shewanella* biofilms focus on their biofilm formation on the anodes of MFCs for electricity generation. Although it is still at proof-of-principle stage, *Geobacter* and *Shewanella* spp. as the two best studied groups of exoelectrogens also show the promise to grow on the cathodes for the synthesis of valuable chemicals by using electrode-feeding electrons as reducing power and CO_2_ as a feedstock (i.e., electrobiosynthesis) (Dumas et al., 2008; Ross et al., 2011; Soussan et al., 2013; Ueki et al., 2018). This technology is attractive for both the alleviation of the world demands for fossil-based fuel and the decrease of CO_2_ emissions. The future research should also focus on the *Geobacter* and *Shewanella* biofilms on the cathodes. Previous studies demonstrated that both *Geobacter* and *Shewanella* cells express different cytochromes and regulate metabolism pathways when they are grown at different electrode potentials (Levar et al., 2014; Hirose et al., 2018). Thus, one of future directions is to investigate the mechanism of electron transfer from cathodes to the cells. Furthermore, the strategies to engineer *Geobacter* and *Shewanella* biofilms formed on the cathodes for the enhanced performance of electrobiosynthesis should also be explored.
